# Topographic Mapping of the Primary Sensory Cortex Using Intraoperative Optical Imaging and Tactile Irritation

**DOI:** 10.1007/s10548-022-00925-w

**Published:** 2022-11-29

**Authors:** Witold H. Polanski, Martin Oelschlägel, Tareq A. Juratli, Hannes Wahl, Pawel M. Krukowski, Ute Morgenstern, Edmund Koch, Gerald Steiner, Gabriele Schackert, Stephan B. Sobottka

**Affiliations:** 1 Department of Neurosurgery, University Hospital of Dresden, Fiedlerstr. 74, 01307 Dresden, Germany; 2 Clinical Sensoring and Monitoring, Faculty of Medicine, University Hospital Carl Gustav Carus, Dresden University of Technology, 01307 Dresden, Germany; 3 Institute of Diagnostic and Interventional Neuroradiology, University Hospital Carl Gustav Carus, Technical University of Dresden, Fetscherstr. 74, 01307 Dresden, Germany; 4 Institute of Biomedical Engineering, Faculty of Electrical and Computer Engineering, Technische Universität Dresden, 01307 Dresden, Germany

**Keywords:** Intraoperative optical imaging, Functional MRI, Sensory irritation

## Abstract

The determination of exact tumor boundaries within eloquent brain regions is essential to maximize the extent of resection. Recent studies showed that intraoperative optical imaging (IOI) combined with median nerve stimulation is a helpful tool for visualization of the primary sensory cortex (PSC). In this technical note, we describe a novel approach of using IOI with painless tactile irritation to demonstrate the feasibility of topographic mapping of different body regions within the PSC. In addition, we compared the IOI results with preoperative functional MRI (fMRI) findings. In five patients with tumors located near the PSC who received tumor removal, IOI with tactile irritation of different body parts and fMRI was applied. We showed that tactile irritation of the hand in local and general anesthesia leads to reliable changes of cerebral blood volume during IOI. Hereby, we observed comparable IOI activation maps regarding the median nerve stimulation, fMRI and tactile irritation of the hand. The tactile irritation of different body areas revealed a plausible topographic distribution along the PSC. With this approach, IOI is also suitable for awake surgeries, since the tactile irritation is painless compared with median nerve stimulation and is congruent to fMRI findings. Further studies are ongoing to standardize this method to enable a broad application within the neurosurgical community.

## Introduction

The determination of exact tumor boundaries within eloquent brain regions is essential to maximize the extent of resection. An accurate preoperative and non-invasive localization of the endangered function, e.g. on the primary sensory cortex (PSC), using anatomical landmarks in CT (Ebeling et al. 1986) or MRI (Berger et al. 1990) or a direct localization by including functional MRI (fMRI) (Boakye et al. 2000; Polanski et al. 2019) is essential to prevent postoperative functional deficits. The integration of the preoperative acquired data into neuronavigation systems allows a safer and more radical surgery around eloquent brain regions (Ganslandt et al. 1999; Nimsky et al. 1999). Intraoperative neurophysiological stimulation and monitoring during tumor surgery near the PSC is the state of the art to further increase patient safety. Somatosensory evoked potentials (SEPs) are widely and routinely used since they were first reported in the mid 1960s by Larson and Sances (Larson and Sances 1966). Recording of the phase reversal of SEPs across the central sulcus allows the identification of the perirolandic gyri (Cedzich et al. 1996). Although widely used, those electrophysiological methods reveal only coarse meshed, punctual information about functional regions during the surgical procedure.

A useful approach to overcome the aforementioned drawbacks is intraoperative optical imaging (IOI) which can be used combined with an adequate stimulation to identify functionally intact peri-tumoral regions of the cortex (Sobottka et al. 2013a, b; Meyer et al. 2013). IOI visualizes differences in cortical optical tissue parameters caused by metabolic changes that correlate well with the neuronal activity (Sheth et al. 2003). Subsequently, it allows the visualization of functional cortex regions as a two-dimensional map by processing the acquired spatio-temporal image data (Raabe et al. 2009). Due to its utility to visualize the extent of the stimulated area on the cortex, IOI allows a topographic functional mapping (Haglund et al. 1992, 1996; Toga et al. 1995; Cannestra et al. 2000, 2004; Lu and Roe 2008; Morone et al. 2017; Sato et al. 2002, 2005, 2017; Nariai et al. 2005; Schwartz et al. 2004; Pouratian et al. 2000). IOI can be used intraoperatively with little preoperative planning or preparation. Compared to intraoperative MRI, IOI is simpler to integrate into the clinical workflow, less time consuming and less cost-intensive in its application. Our own group showed the potential of IOI for the visualization of somatosensory (Sobottka et al. 2013a), visual (Sobottka et al. 2013c), speech and motor (Oelschlagel et al. 2020) areas as well as for the delineation of tumor borders with direct cortical stimulation (Oelschlagel et al. 2018). As for now, we have used IOI to visualize and identify the PSC using a periodic electrical median nerve stimulation in patients under general anesthesia.

However, median nerve stimulation is a painful diagnostic and therefore inapplicable during awake surgeries (Babiloni et al. 2001).

In this technical note, we describe a novel approach of using IOI with painless, direct tactile irritation to demonstrate the feasibility of topographic mapping of different body regions within the PSC. In addition, we compare the IOI results with preoperative fMRI and electrophysiological findings.

## Material and Methods

### Patients

Our study cohort consists of five patients with tumors located near the PSC who received tumor removal. Preoperatively, none of the patients showed permanent sensory deficits. Surgery was performed in general anesthesia in all patients except patient #5 who underwent awake surgery. Patients’ characteristics are described in Table 1.

**Table 1:** IOI imaging hardware

	Hardware setup no. 1	Hardware setup no. 2
Camera	Zeiss AxioCam MRm	Kinevo integrated
Filtering	568 nm	none (RGB camera) evaluation of green channel
Exposure Time	50 ms	1 ms
Microscope	Zeiss OPMI Pico	Zeiss Kinevo
Illumination (microscope integrated)	Xenon 180 W	Xenon 300 W
Resolution	692 × 520 pixel (2 × 2 binning mode)	1920 × 1080 pixel
Digitization	12 bit	8 bit per color channel
Used in Patient	#1–3	#4,5

### Preoperative fMRI

Preoperative 3-Tesla MRI (Siemens Magnetom Verio, Siemens Healthineers, Erlangen, Germany) for neuronavigation and a sensory fMRI were performed for planning of the optimal surgical approach and to visualize the spatial relation between the PSC and the tumor. The preprocessed fMRI data were convolved with the canonical haemodynamic responce function (HRF) using the box-car design, corresponding to the stimulus blocks oft he paradigm. The results were visualised with corrected significance level of p < 0.05 and cluster size of at least 20 Voxel. Thereby, echo-planar imaging (EPI) technics for blood oxygen level dependent (BOLD) sequences were used with an in-plane resolution of 1.6 mm and slice thickness of 3.36 mm. To visualize the cortical sensory region, the hand of the patients was stimulated with a surgical brush scrub during the fMRI acquisition. The stimulation paradigm was a block design with overall 100 scans with 10 scans in resting state following 10 scans with tactile irritation repeated 5 times. Preprocessing was done with SPM12 v7487 running on MATLAB 2018b (MATLAB and Statistics Toolbox Release 2012b, The MathWorks, Inc., Natick, Massachusetts, United States) and consisted of (1) motion correction of the BOLD images, (2) coregistration of the anatomical scan to the mean BOLD image and (3) smoothing of the BOLD images with an 8 mm Gaussian kernel. Default settings were used for model specification.

### IOI

IOI was performed in the patients after the craniotomy, before resection. Two different hardware setups were used (see Table 1). The setup no. 1 consists of a charge-coupled device camera (AxioCam MRm, Carl Zeiss MicroImaging, Jena, Germany) attached via a beam splitter to a surgical microscope. Light filtering was performed using a bandpass interference filter (Edmund Optics, Barrington, USA) with a central transmission wavelength of at λ = 568 nm aiming at changes in regional cerebral blood volume (ΔCBV). For the measurements in patient #4 and patient #5 images were acquired with the RGB camera integrated into the surgical microscope (Zeiss Kinevo, Carl Zeiss Meditec AG, Oberkochen, Germany). The video files were in these cases postoperatively processed using the same Fourier-based image processing pipeline that was used for images acquired with hardware setup no. 2 as described in detail elsewhere (Oelschlagel et al. 2013). In short, a pixelwise FFT was performed, and the resulting amplitude/power spectral density information at stimulation frequency (${f}_{stim} = 1/60 \mathrm{Hz}$) was used for calculation of a two-dimensional map (P_s_). This map, derived from stimulation frequency was then divided by second a two-dimensional map that was derived from the sum of the different vasomotion frequency components (P_VLF_) within the range ${f}_{VLF}< 0.04 Hz$. Therefore, the P_s_/P_VLF_ that are shown here, are representing the percentage of the activation frequency in respect to the vasomotor very-low frequency bands. Those maps are reliable able to identify functional areas within the PSC, as shown in prior publications (Sobottka et al. 2013a; Meyer et al. 2013).

### Electrophysiological Measurements and Stimulation Protocols

After neuronavigation-guided craniotomy and durotomy, phase reversal of somatosensory evoked potentials was measured at the exposed brain cortex to identify the SC. Afterwards, the median nerve at the wrist of the patients was electrical stimulated for visualization of the corresponding activated cortical region. A Bravo/Endeavor neurostimulator (Nicolet Biomedical, Madison, WI, USA) was used with a stimulation current of 20 mA and a stimulation frequency of 5.1 Hz. For each stimulation site, image data of 9 cycles with a 30 s. rest period and a 30 s. stimulation period were acquired from the cortical surface.

In all patients the hand region was brushed with a surgical rubber for tactile irritation. In addition, in patient #3 the arm and leg areas were brushed to study the topographic distribution.

### Comparison of fMRI and IOI

**Fig. 1:**
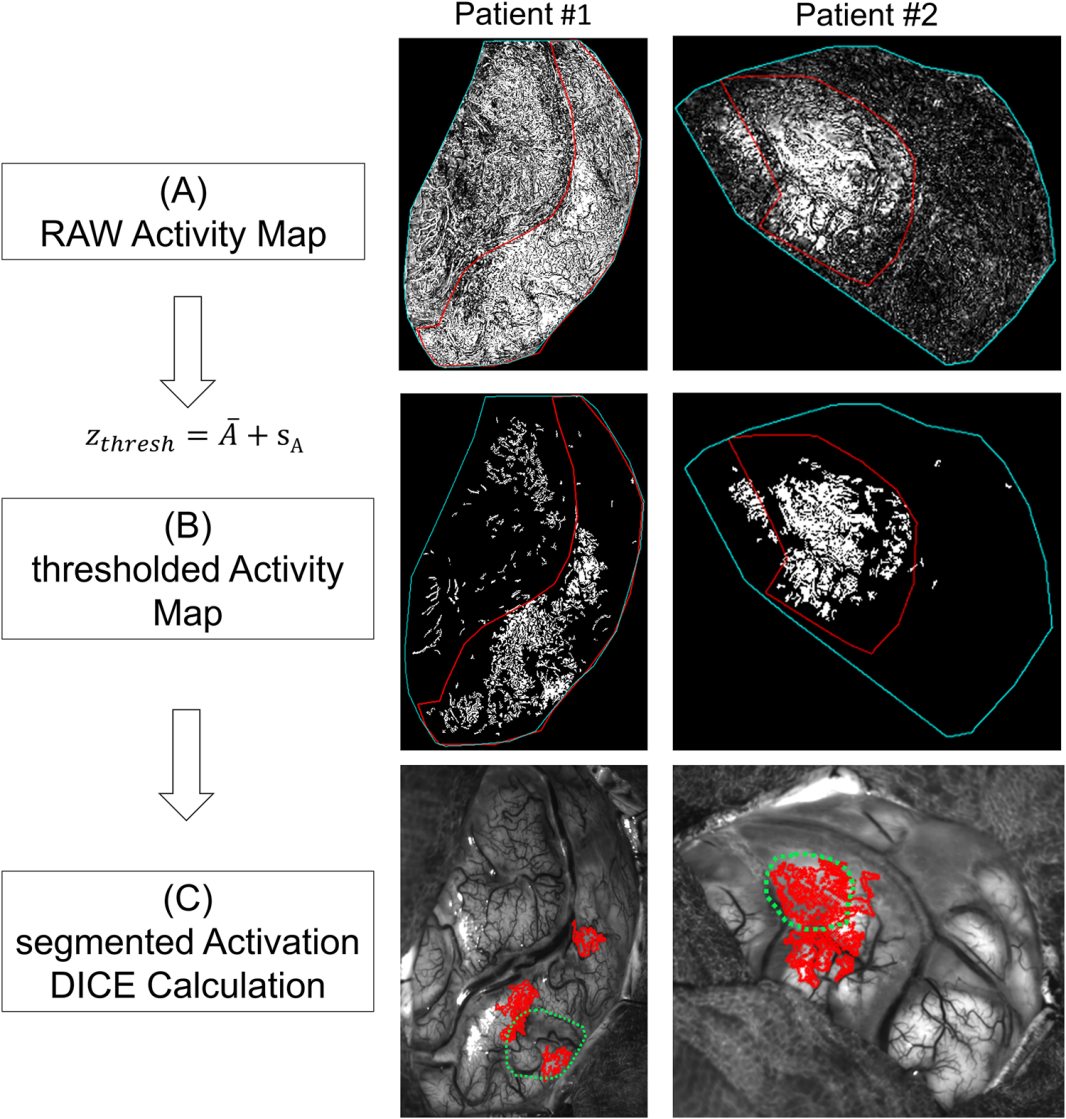
Image processing for comparison of IOI and fMRI activations. **A** Overlay of segmentation borders for PSC (red line) and trepanation (blue line) over raw activity map. **B** Thresholded and morphologically processed activity maps. Threshold values: patient #1 z_thresh_ = 0.12; patient #2 z_thresh_ = 0.23 **C** Segmented IOI as well as fMRI activations for which the DICE coefficient calculation was performed

In patient #1 and patient #2 a comparison of the location of IOI and fMRI activation was performed. Therefore, the location of fMRI activation on PSC as well as the PSC itself was manually segmented within visible light image of IOI (green dotted line/red line, see Fig. 1). Subsequently, the IOI raw activity map was thresholded using a threshold ${z}_{thresh }=\overline{A }+{s}_{A}$ whereas $\overline{A }$ is the mean value of the activity map within the trepanned area and ${s}_{A}$ the corresponding standard deviation. The resulting binary map was morphological processed by removing all connected components smaller 10 pixel (opening). Finally, the three largest connected areas were segmented and treated as IOI activation area. The DICE coefficient, a statistical tool that measures the similarity between two sets of data, was calculated between this IOI activation and the segmentation of the fMRI activation. Furthermore, the DICE coefficient of the convex hull of the activation as well as DICE coefficient for the complete thresholded IOI activity map (see Fig. 1B) was taken into consideration. The distributions of the pixel values from the raw activity map within the different regions segmented during this process were compared.

## Results

In all five patients, a well delineated activity within the IOI amplitude maps was visible using two different hardware setups; The RGB imaging (patients #4, 5) delivered similar activations by evaluation of the green channel compared with the CBV maps at 568 nm (patients #1–3). Moreover, the IOI activity maps correlated well with electrophysiological measurements and anatomical landmarks located on the PSC.

Notably, since patient #5 was recorded under local anesthesia, a higher visualization threshold was needed for a well delineated activation than the threshold used for the patient under general anesthesia. The results for each patient recorded with IOI and stimulated by tactile irritation are shown in Table 2. In patients #1–4, IOI was performed additionally with median nerve stimulation.

**Table 2:** Patient’s demographics and tumor characteristics

Patient	#1	#2	#3	#4	#5
Sex	Male	Female	Female	Male	Female
Age	81 years	78 years	32 years	54 years	32 years
Tumor location	Left precentral cortex	Left parietal posterior to the postcentral gyrus	Left parasagittal next to the postcentral gyrus	Left parietal posterior to the postcentral gyrus	Right precentral cortex
Histological diagnosis	Metastasis of a malignant melanoma	Metastasis of an adenocarcinoma of the lung	Fibrous meningioma WHO grade 1	Glioblastoma multiforme	Anaplastic glioma
Preoperative symptoms	Right hand paresis	Epileptic seizure	Epileptic seizure	Epileptic seizure	Left hand paresis
Anaesthesia	General	General	General	General	Local
3D approach reconstruction					
IOI					

A 3D approach reconstruction in each case is shown together with amplitude maps of intraoperative optical imaging for the tactile irritation of the hand. Dotted line marks the central sulcus. The magenta area represents the superficial tumor localization, while the magenta arrow marks the starting point for the trans-sulcal approach towards deeper located tumors

**Fig. 2:**
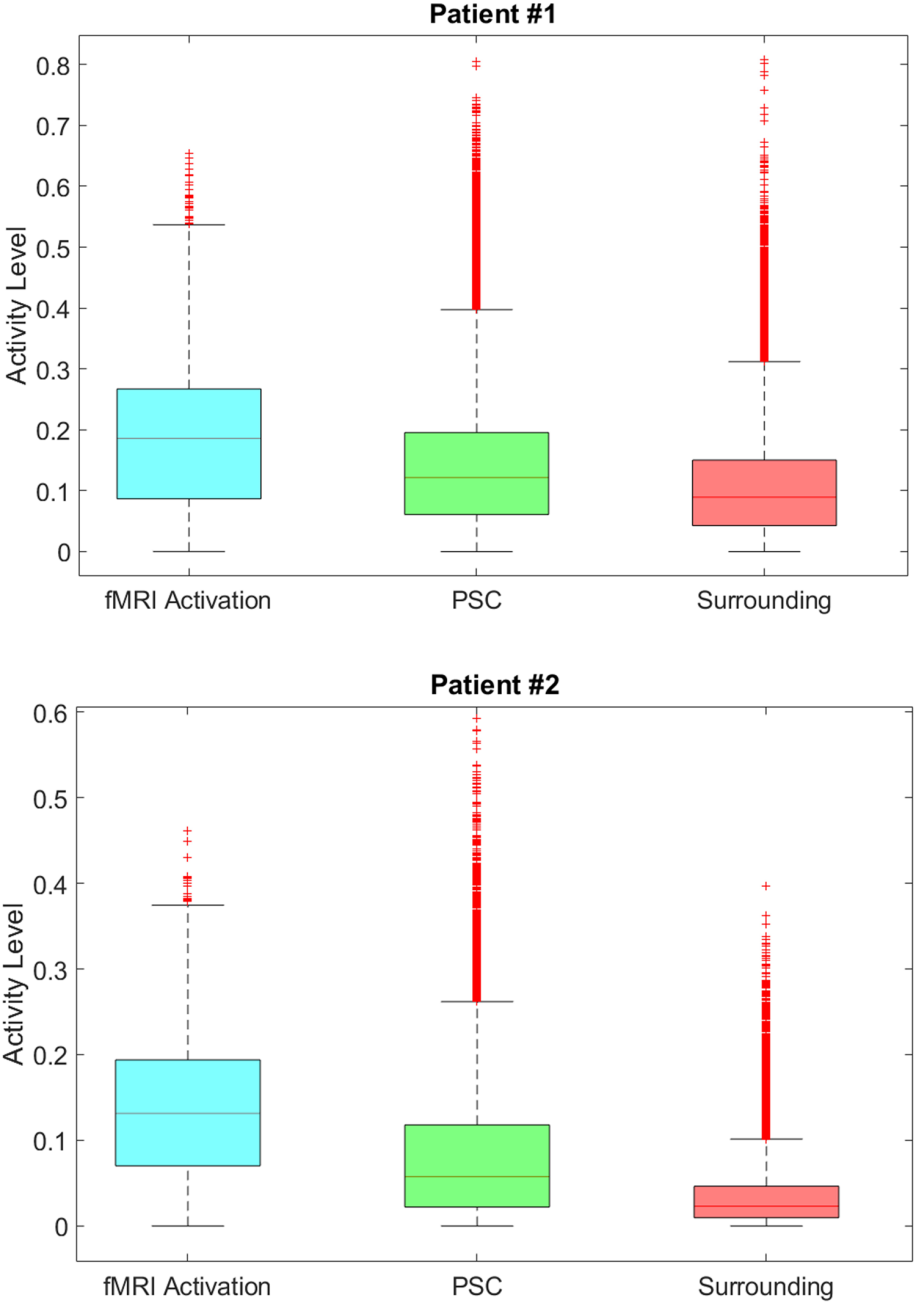
Pixel values of activity map within the different segmented regions. The regions are in correspondence to Fig. 1C. The PSC labeled region does not include the region of fMRI activation and the Surrounding labeled region is correlating with all pixels of trepanation except the PSC region. Median/Q1/Q3 for the 3 regions (left to right of boxplot) are as following: Patient#1 0.19/0.09/0.27; 0.12/0.06/0.20; 0.09/0.04/0.15; Patient #2 0.13/0.07/0.19; 0.06/0.02/0.12; 0.02/0.01/0.05

**Fig. 3:**
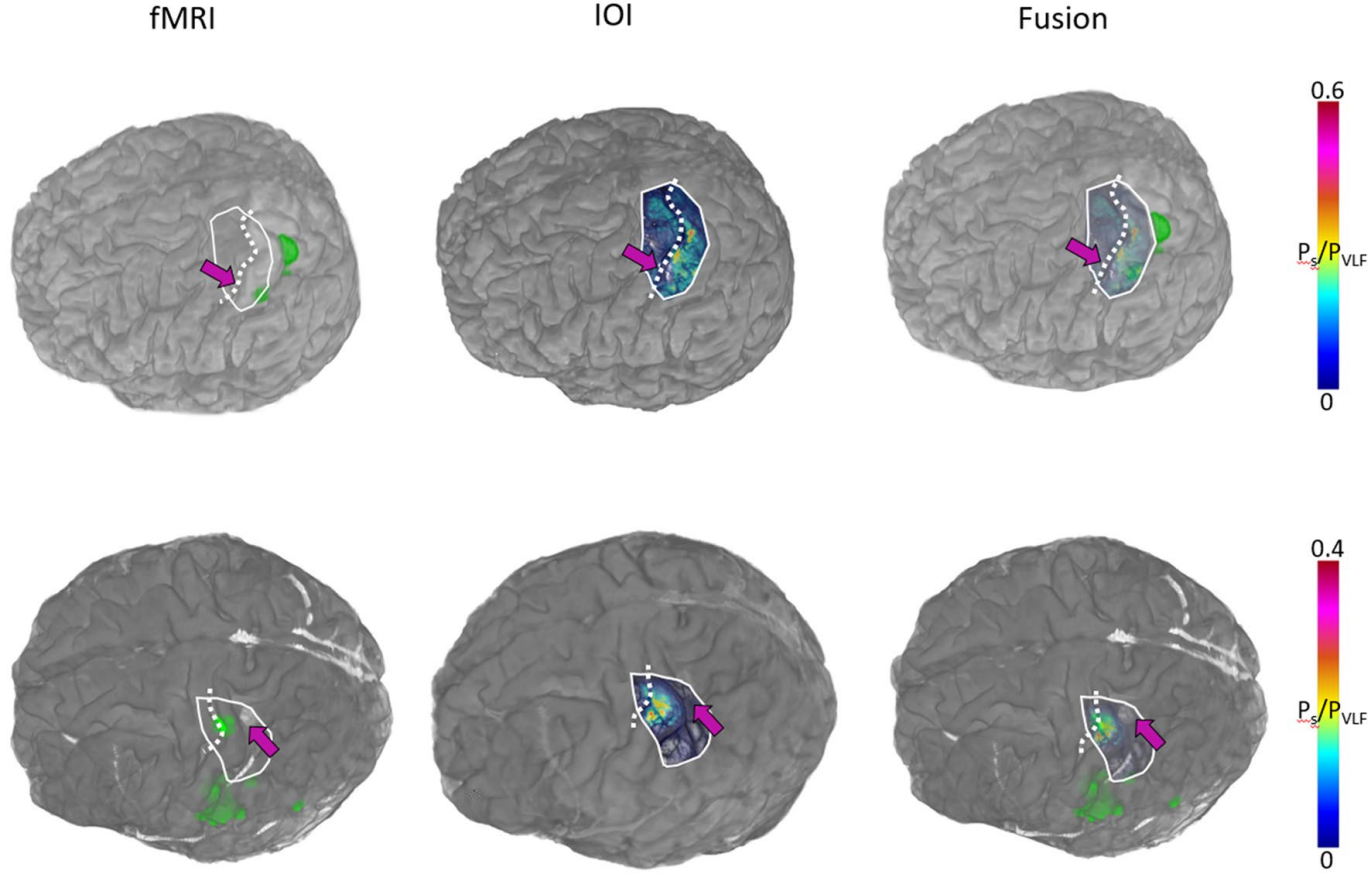
Activation following tactile irritation for different imaging modalities in patient #2: fMRI (left), intraoperative optical imaging (IOI, middle) and fusion of both methods (right). The magenta arrow indicates the starting point for the transsulcal approach towards deeper located tumors. The dotted line marks the central sulcus. Fusion of both modalities was created by registering manually the IOI whitelight image and the fMRI based on anatomical landmarks. Subsequently, the IOI image was semi-transparent overlaid to the fMRI dataset

Comparing IOI and the preoperative fMRI with tactile irritation in patients #1, 2 (see Figs. 1, 2, 3), we saw a good visual correlation between the imaging modalities. The DICE coefficient for IOI and fMRI activation is $DSC = 0.18/0.48/0.26$ (patient #1, fMRI & IOI activation/fMRI & convex hull over IOI activation/fMRI & complete thresholded IOI map within trepanned region) respectively $DSC = 0.53/0.61/0.45$ (patient #2). The quantitative comparison of the single pixel activity levels (P_s_/P_VLF_) for the different regions of the activity maps for the two patients (Fig. 2), reveals substantial higher values within the regions of the IOI maps, that were segmented as areas, being also in fMRI activated. Those fMRI regions showed also a higher median activity level when compared to the whole PSC as well as to the surrounding area.

**Fig. 4:**
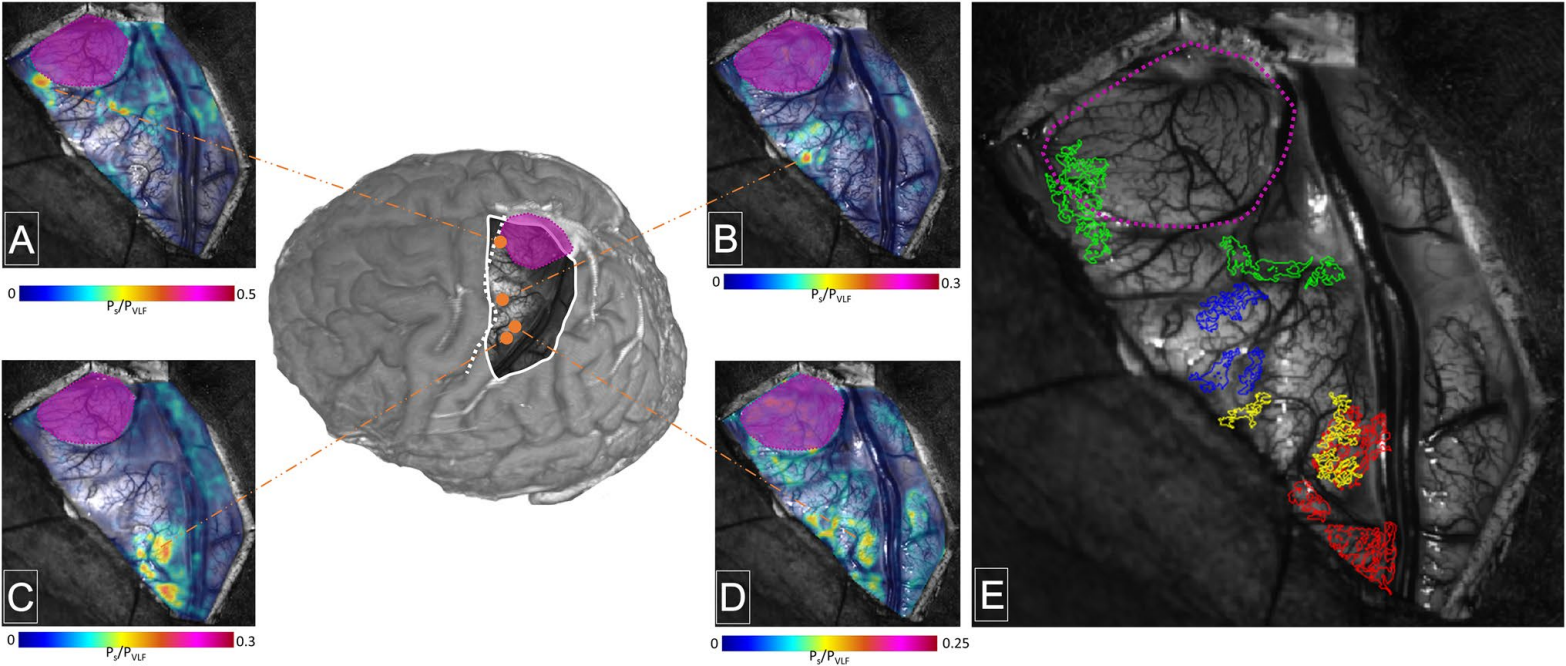
Topographical mapping of activated cortical regions after tactile irritation of the leg (**A**), the upper arm (**B**) and the hand (**D**) and after electrical stimulation of the median nerve (**C**) in patient #3. The magenta area represents the cortical part of the tumor localization. **E** shows the segmented activation centers (methodology see Fig. 1) for the different body parts (green = leg, blue = upper arm, yellow = hand tactile, red = hand electrical median nerve stimulation)

The tactile irritation of different body areas—including the hand region—in patient #3 revealed a plausible topographic distribution along the PSC, which allows a fine cortical distinction of different body regions (Fig. 4). In addition, in this case (patient #3), we compared the median nerve stimulation at the wrist with the tactile irritation of the hand (Fig. 4C–E). Hereby, the median nerve stimulation revealed a more delineated and compact area, whereas the tactile irritation was associated with a more diffuse activation, although parts of activation between both stimulation modalities are in very good agreement ($DSC=0.16/0.28/0.09$; IOI activations tactile & electrical/convex hull over IOI activations tactile & electrical/complete thresholded IOI maps tactile & electrical within trepanned region).

All five patients received after measurements a complete tumor resection with preservation of the PSC function. No complications occurred and no further neurological declines was observed after surgery. During the 9 min measurement with 9 cycles of 30 s. rest and 30 s. stimulation, the active and passive phase were clearly displayed within the software, while the post-processing phase took a few seconds. After the measurement, the surgeon in charge was able to modify the threshold to optimize the image quality immediately. Adverse events related to stimulation or tactile irritation were not observed.

## Discussion

In this study we showed that tactile irritation of the hand in local and general anesthesia is associated with reliable changes of cerebral blood volume during IOI. Due to the fact that electrical stimulation is painful, it is not applicable during awake surgeries. Here, we demonstrate that IOI activation maps induced by painless tactile irritation were comparable with median nerve stimulation. Our findings highlight the feasibility of tactile irritation of different body regions as an adjunct tool with IOI for the visualization of different sensory processing areas. Most importantly our approach was successful even when performed in patients under general anesthesia. Due to the instantly computed visualization maps, we envision that our approach might be helpful in intraoperative decision makings regarding tumor removal.

Several groups have demonstrated that tactile irritation leads to a reliable activation of PSC using fMRI (Stippich et al. 1999; Kalberlah et al. 2013; Schweizer et al. 2008; Chen et al. 2002; Disbrow et al. 1998). Indeed, we observed a good visual correlation between IOI and the preoperative fMRI with tactile irritation.

Moreover, few is known about tactile irritation during IOI. In a recent work, Caredda et al. used IOI with an RGB camera to evaluate functional areas while inducing tactile irritation in a single case (Caredda et al. 2019).

Of note, before starting the measuring phase of IOI, the surgeon has to be aware of several factors that might influence the image quality (e.g. light intensity changes during measurement, changes of the cortex temperature due to rinse with water, blood in the region of interest) (Sobottka et al. 2013b). A further difficulty is to position the microscope without any artifacts caused by specular reflections. Furthermore, no relevant preoperative sensory deficits should be present in candidate patients for IOI. Otherwise, we suppose that tactile irritation for IOI might be not successful for visualization of the corresponding PSC areas, as proven for electrical stimulation in previous studies (Sobottka et al. 2013b).

Additionally, compared with electrical stimulation, tactile irritation could be associated with more motion artifacts that need to be compensated in the post processing phase. Consequently, we applied a non-rigid registration algorithm to address these artifacts. Furthermore, using a new IOI hardware setup with a microscope integrated RGB camera, we were able to generate activity maps with good quality. The physiological origin of the signal is much more difficult to interpret in cases acquired with the RGB setup. Due to the broadband filtering of the green camera channel, fractional changes of oxy- and deoxyhemoglobin as well as blood volume changes are contributing in different weightings towards the measured reflectance change. However, further research is needed to assess the use of an RGB camera without light filter.

## Conclusions

The combination of IOI with tactical irritation leads to a reliable topographical visualization of functional cortical areas in the PSC. Thereby, we saw a good visual correlation between IOI and the preoperative fMRI with tactile irritation. With this approach, IOI is also suitable for awake surgeries, since the tactile irritation is painless compared with median nerve stimulation.

Further studies are ongoing to standardize this method to enable a broad application within the neurosurgical community.
